# Definitions and drivers of relapse in patients with schizophrenia: a systematic literature review

**DOI:** 10.1186/1744-859X-12-32

**Published:** 2013-10-23

**Authors:** José M Olivares, Jan Sermon, Michiel Hemels, Andreas Schreiner

**Affiliations:** 1 Department of Psychiatry, Hospital Meixoeiro, Complejo Hospitalario Universitario de Vigo, Vigo 36200, Spain; 2 Janssen-Cilag NV/SA, Antwerpseweg 15-17, Beerse 2340, Belgium; 3 Janssen Health Economics Market Access and Reimbursement, Europe, Middle East and Africa, Hammerbakken 19, Birkerød 3460, Denmark; 4 Medical and Scientific Affairs, Janssen-Cilag Europe, Middle East and Africa, Johnson & Johnson Platz 5a, Neuss 41470, Germany

**Keywords:** Relapse, Hospitalization, Schizophrenia, Definition, Adherence, Drivers

## Abstract

Relapse in patients with schizophrenia has devastating repercussions, including worsening symptoms, impaired functioning, cognitive deterioration and reduced quality of life. This progressive decline exacerbates the burden of illness on patients and their families. Relapse prevention is identified as a key therapeutic aim; however, the absence of widely accepted relapse definition criteria considerably hampers achieving this goal. We conducted a literature review in order to investigate the reporting of relapses and the validity of hospitalization as a proxy for relapse in patients with schizophrenia. The primary aim was to assess the range and validity of methods used to define relapse in observational or naturalistic settings. The secondary aim was to capture information on factors that predicted or influenced the risk of relapse. A structured search of the PubMed database identified articles that discussed relapse, and hospitalization as a proxy of relapse, in patients with schizophrenia. National and international guidelines were also reviewed. Of the 150 publications and guidelines identified, 87 defined relapse and 62% of these discussed hospitalization. Where hospitalization was discussed, this was as a proxy for, or a component of, relapse in the majority of cases. However, hospitalization duration and type varied and were not always well defined. Scales were used to define relapse in 53 instances; 10 different scales were used and multiple scales often appeared within the same definition. There were 95 references to factors that may drive relapse, including non-adherence to antipsychotic medication (21/95), stress/depression (11/95) and substance abuse (9/95). Twenty-five publications discussed the potential of antipsychotic therapy to reduce relapse rates—continuous antipsychotic therapy was associated with reduced frequency and duration of hospitalization. Non-pharmacological interventions, such as psychoeducation and cognitive behavioural therapy, were also commonly reported as factors that may reduce relapse. In conclusion, this review identified numerous factors used to define relapse. Hospitalization was the factor most frequently used and represents a useful proxy for relapse when reporting in a naturalistic setting. Several factors were reported to increase the risk of relapse, and observation of these may aid the identification of at-risk patients.

## Introduction

Schizophrenia is a highly prevalent disorder affecting approximately 1% of the world's population [1]. Patients with schizophrenia often require antipsychotic medication throughout their lifetime. With correct management, many patients can achieve symptomatic remission [2] as defined, for example, by the Andreasen remission criteria [3]. However, relapses are a highly prevalent component of the disease course [4,5]. Schizophrenia is a significant burden, for both patients and families. The clinical deterioration associated with each subsequent relapse only serves to exacerbate this. For patients, relapse can have devastating repercussions such as worsening of symptoms, progressive cognitive deterioration, impaired functioning and reduced quality of life [6-9]. Furthermore, families are affected by the emotional stress and financial burden of living with and caring for a patient with schizophrenia [10,11]. To minimize the burden on the patient and family, it is vital that periods of effective symptom control are extended for as long as possible.

International guidelines identify relapse prevention as a key therapeutic aim [4-6]; however, there are currently no established criteria by which to define relapse, and our current understanding of relapse may not be sufficient to combat this problem effectively [6,7,12,13].

We conducted a structured literature search to investigate the reporting of relapse and the validity of hospitalization as a proxy for relapse in patients with schizophrenia. Additionally, the factors that may be used to predict a relapse and those factors associated with increased or decreased risk of relapse were also investigated.

## Methods

### PubMed search and literature review

A structured search of the National Center for Biotechnology Information (NCBI) PubMed database was performed to identify articles published from 1 January 2000 to 1 May 2010 that discussed relapse, and hospitalization as a proxy for relapse, in patients with schizophrenia. The search term ‘Schizophrenia [Title/Abstract] AND ((Relapse[Title/Abstract]) OR (Hospitalization[Title/Abstract]))’ and limits (Humans, Clinical Trial, Meta-Analysis, Review, Classical Article, Clinical Trial, Phase IV, Comparative Study, Congresses, Controlled Clinical Trial, Corrected and Republished Article, Evaluation Studies, Government Publications, Journal Article, Multicenter Study, Published Erratum, English, French, German, Italian, Spanish) were used to identify relevant literature from the database. The latest national (France, Spain, Germany, Italy and the UK) and international (World Federation of Societies of Biological Psychiatry (WFSBP); American Psychiatric Association (APA)) guidelines were also reviewed. The primary objective of this literature search was to determine whether specific criteria have been used to define relapse in observational and naturalistic settings. The secondary aim was to identify possible factors that may drive or reduce relapse.

Randomized clinical trials (RCTs) represent highly controlled situations in which patients are required to meet stringent inclusion criteria. Furthermore, subjects are frequently randomized to a fixed medication dose, without provision for dose optimization to meet individual requirements, which may likely have an impact on patient outcomes. Patients are closely monitored in RCTs, and specific relapse definitions are imposed for consistency across study centres. Hence, RCTs are less informative in regard to routine definitions of relapse used in clinical practice and to factors associated with relapse in unselected clinical populations. Therefore, data from primary publications of randomized, placebo-controlled clinical trials of pharmacological agents were excluded from this literature search.

### Abstract review

Identified abstracts were excluded if they were not relevant to one of the four domains:

1. Definition of relapse.

2. Factors that may predict, drive or reduce relapse.

3. Duration and frequency of relapse.

4. Cost and resource use associated with relapse.

Guidelines were reviewed using the same criteria as the abstracts identified in the PubMed search. Following this review, the selection of literature included 206 abstracts and five guidelines.

Publications discussing the definition of relapse and factors that may predict, drive or reduce relapse are reviewed here. Those discussing the duration and frequency of relapse or associated cost and resource are reviewed elsewhere.

### Full article review

Following abstract review, each corresponding full article was reviewed and excluded if not relevant to one of the four domains above. Following this review stage, the selection of literature included 156 journal papers and five guidelines. A final review of the literature was conducted. During this final review, the authors excluded those papers describing cost-effectiveness modelling studies that constituted secondary research as accurate interpretation and evaluation of such papers require an in-depth analysis of the methodologies and assumptions used, which was deemed to be beyond the scope of the current review.

**Figure 1:**
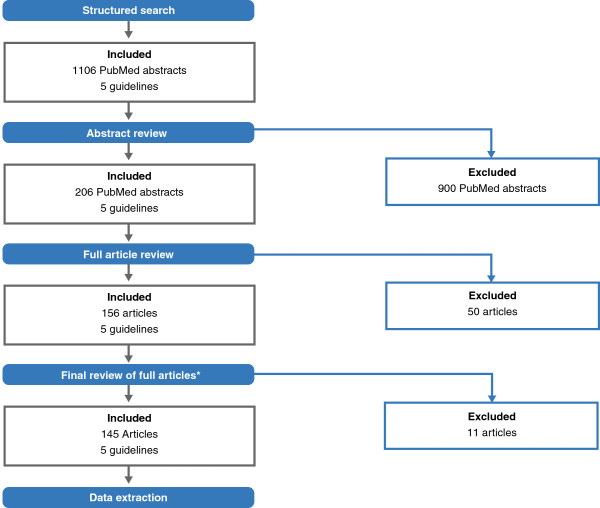
**Literature search process.** The *asterisk* denotes that the final review process is described in detail in the main body of the text.

The final literature selection included 145 journal manuscripts and five guidelines from three organisations (APA [6,14], National Institute for Clinical Excellence (NICE) [4] and WFSBP [2,5]). Figure 1 details the full literature search process.

## Results

### Definitions of relapse

**Figure 2:**
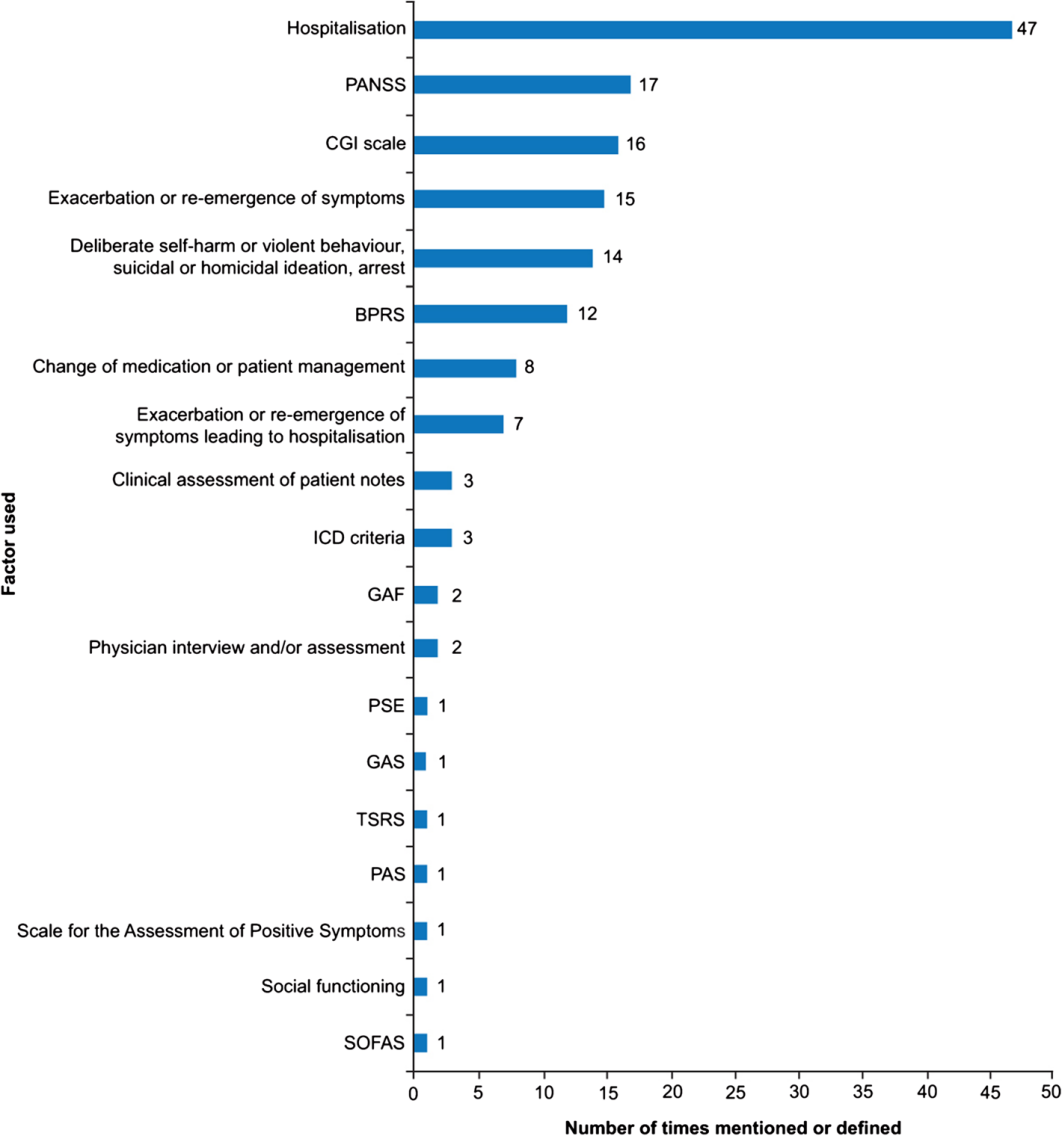
**Reported components of the definition for relapse.** Hospitalization [11,15-59]; Positive and Negative Syndrome Scale (PANSS) [7,15,17,18,60-72]; Clinical Global Impression (CGI) scale [17,18,26,30,52,57,60-62,65],[66,68,71,73,74]; exacerbation/re-emergence of symptoms [7,27,29,34,38,43,63,67],[75-81]; deliberate self-harm or violent behaviour, suicidal or homicidal ideation, arrest [18,23,27,43,49,50,57,65],[66,71,74,82-84]; Brief Psychiatric Rating Scale (BPRS) [28,43,71,76,84-91]; change of medication or patient management [18,27,38,41,56,66,75,92]; exacerbation/re-emergence of symptoms leading to hospitalization [20,66,92-96]; clinical assessment of patient notes [38,57,88]; International Classification of Diseases (ICD) criteria [70,89,97]; Global Assessment of Functioning (GAF) [64,72]; physician interview and/or assessment [86,98]; Present State Examination (PSE) [84]; Global Assessment Scale (GAS) [84]; Target Symptoms Ratings Scale (TSRS) [76]; Psychiatric Assessment Scale (PAS) [99]; scale for the assessment of positive symptoms [86]; social functioning [75]; Social and Occupational Functioning Assessment Scale (SOFAS) [60].

Of the final selection of literature, 87 manuscripts included a definition of relapse. None of the identified guidelines defined relapse. Figure 2 describes the factors used alone or in combination to define relapse. Hospitalization was the most widely used factor as a proxy for relapse or as a component of the definition.

#### Hospitalization

‘Hospitalization’ appeared in 54% (47/87) of the publications that defined relapse, 62% (54/87) if grouped with ‘exacerbation of symptoms leading to hospitalization’. In these 54 publications, hospitalization or exacerbation of symptoms leading to hospitalization was discussed in 56 separate instances. In the majority (55%, 31/56) of cases, hospitalization was used as a direct proxy for relapse or as a component of a relapse definition. In the remainder (45%, 25/56), hospitalization was discussed independently or without reference to relapse (Table 1). The majority of publications did not define the length of the hospitalization or the type of hospitalization—generic terms of ‘hospitalization’ and ‘psychiatric hospitalization’ were predominantly used. Two publications, published by the same authors, defined hospitalization as ‘36 h of full hospitalization or a 5-day partial hospitalization due to an exacerbation of acute psychotic symptoms’. However, the difference between ‘partial’ and ‘full’ hospitalization was not defined in these publications [15,16].

**Table 1:** Manuscripts that defined relapse and also discussed hospitalization used a range of definitions

**Way in which hospitalization is used in references that also define relapse**	**Number of times hospitalization defined ( *n *)**	**References**
Hospitalization is discussed, separately to relapse	25	Bechdolf et al. [16]; Crown et al. [59]; dos Reis et al. [47]; Dyck et al. [32]; Hayhurst et al. [33]; Miettunen et al. [40]; Olivares et al. [56]; Svarstad et al. [31]; Sun et al. [45]; Rabinowitz et al. [30]; Taylor et al. [22]; Thompson et al. [36]; Usall et al. [37]; Ward et al. [25]; Weiden et al. [26]; Whitehorn et al. [39]; Janicak et al. [48]; Zhu et al. [50]; Bechdolf et al. [15]; Drake et al. [20]; Novák-Grubic and Tacvar [34]; Olivares et al. [23]; Valencia et al. [46]; Kim [54]; Peuskens et al. [24]
Hospitalization equates to relapse	10	Buckley et al. [44]; de Sena et al. [35]; Hawley et al. [58]; Leucht et al. [30]; Malik et al. [11]; Rouillon et al. [49]; Spaniel et al. [52]; Spaniel et al. [53]; Taylor et al. [22]; Turkington et al. [42]
Hospitalization used as a component of relapse definition	14	Almond et al. [38]; Ascher-Svanum et al. [57]; Csernansky et al. [17]; Drake et al. [20]; Emsley et al. [18]; Gaertner et al. [28]; Gasquet et al. [55]; Haro et al. [21]; Haro et al. [51]; Hickling et al. [29]; Hong et al. [19]; Muirhead et al. [41]; Pharoah et al. [27]; Xiang et al. [43]
Exacerbation of symptoms leading to hospitalization equates to relapse	7	Berglund et al. [95]; Chabannes et al. [96]; Drake et al. [20]; Lancon et al. [93]; Simpson et al. [66]; Tomaras et al. [92]; Wahlbeck et al. [94]

#### Scales

There were 53 instances of a scale being used to define relapse; however, multiple scales often appeared within the same definition. Ten different scales were used to define relapse (Figure 2), including the Positive and Negative Syndrome Scale (PANSS), Clinical Global Impression (CGI) scale (including the CGI-Severity, CGI-Schizophrenia and CGI-Change subscales), Brief Psychiatric Rating Scale (BPRS) and Global Assessment of Functioning (GAF) scale, the most frequently cited being PANSS and CGI. Generally, the scales used to define relapse assessed symptom severity and in particular the positive symptoms of schizophrenia.

There was considerable variation between studies in the use of each scale, in terms of the thresholds applied, and subscales used within the relapse criteria. Among the 18 instances of using the PANSS to define relapse, thresholds included an overall increase in the scale [7,60,61], an increase in the score from baseline [17,18] and recording of a score >4 for certain individual PANSS items [62-64]. There was wide variation between studies when using the CGI scale, since many different subscales and thresholds were proposed to define relapse. CGI-Severity (CGI-S) was the most commonly cited subscale, with CGI-schizophrenia (CGI-SCH) and CGI-Change (CGI-C) also frequently used. However, the threshold for relapse was broadly similar regardless of the CGI subscale used—with an overall increase (or increase in a single factor) to a score of 6 or 7 being the most frequent measure [18,19,60,65,66].

#### Other definitions

Sixteen per cent (14/87) of publications defined relapse as a change in behavioural patterns towards more violent or self-destructive ideation or tendencies. Exacerbation or re-emergence of symptoms was the fourth most common component of definitions identified in the literature search, second if combined with those exacerbations that led to hospitalization.

### Factors that may increase or decrease the risk of relapse

Ninety-four journal articles and five guidelines discussed the various factors that may drive or reduce relapse rates in patients with schizophrenia.

#### Factors that may drive relapse

**Figure 3:**
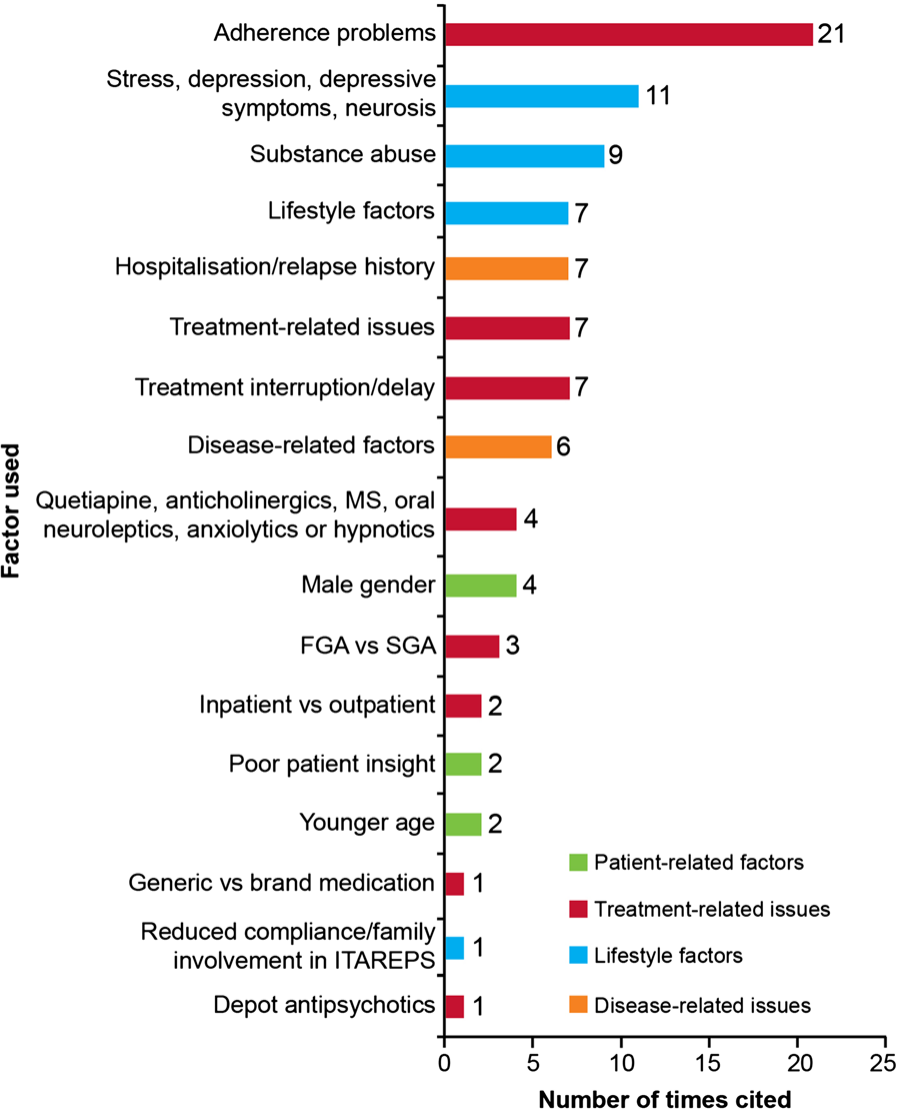
**Potential drivers of relapse.** Adherence problems [2,6,7,13,17,25,26,31],[34,45,47,53,57,76,85,100-105]; stress, depression, depressive symptoms, neurosis [2,6,7,12,55,59,70,97],[106-108]; substance abuse [2,6,7,21,31,57,59,83],[97]; lifestyle factors [7,12,21,38,79,109,110]; hospitalization or relapse history [21,31,38,40,57,59,97]; treatment-related issues [6,7,28,43,61,70,111]; treatment interruption or delay [2,6,12,75,85,112,113]; disease-related factors [57,70,83,85,97,114]; quetiapine, anticholinergics, mood stabilizers (MS), oral neuroleptics, anxiolytics or hypnotics [21,31,51,55]; male gender [30,37,83,115]; use of first-generation antipsychotics (FGA) vs second-generation antipsychotics (SGA) [30,85,116]; outpatient vs inpatient [29,39]; poor patient insight [20,83]; younger age [57,83]; generic vs branded medication [117]; reduced compliance/family involvement in Information Technology Aided Relapse Prevention in Schizophrenia (ITAREPS) programme [53]; depot antipsychotics [76].

There were 95 references to factors that may drive relapse (Figure 3), with non-adherence to antipsychotic medication the most frequently reported factor. For example, in a study of first-episode patients, medication non-adherence was observed in 70% of patients with relapse, compared with only 25% of those without relapse, at 1-year follow-up (*χ*^2^ = 11.2, *p* = 0.001) [85]. Furthermore, in patients with recently diagnosed (≤ 2 years) schizophrenia, a 69% relapse rate was observed in patients non-adherent to oral or depot antipsychotic therapy compared with a rate of 18% in adherent patients (*χ*^2^ = 12.66, *p* < 0.001) [20].

Patient-specific, lifestyle and disease-related factors associated with increased rates of relapse were also identified in the search (Figure 3). Stress/depression and substance abuse were the second and third most frequently reported factors associated with relapse. In a retrospective cohort study, depression (adjusted hazard ratio (AHR) = 1.44; 95% CI = 1.05, 1.98; *p* < 0.05) and substance abuse (AHR = 1.80; 95% CI = 1.32, 2.47; *p* < 0.05) were significantly associated with an increased risk of psychiatric hospitalizations [97].

There were 46 instances where treatment-related factors, such as side effects, dosing issues, efficacy and generic antipsychotic use, were associated with increased relapse rates (Figure 3). Delay in treatment delivery and interruptions to treatment due to loss of medical insurance coverage were also identified. In one study, a longer duration of untreated psychosis was significantly associated with mild relapse in the first year after hospitalization (Kruskal-Wallis test *χ*^2^ = 5.31, *p* = 0.02). In this study, a mild relapse was defined as a recurrence or exacerbation of psychotic symptoms in 1 week for which an increase in antipsychotic medication was required, without a significant decline in social functioning [75]. A regression model analysis in another study indicated that patients who were subject to interruptions in Medicaid coverage were more likely to be hospitalized (negative binomial regression *z* = 6.9, *p* < 0.001), experience a greater number of hospitalizations (negative binomial regression *z* = 8.4, *p* < 0.001) and spend 61% more days in the hospital compared with patients with continuous coverage (exponential regression model *z* = 7.1, *p* < 0.001) [112].

#### Factors that may reduce the rate of relapse

**Figure 4:**
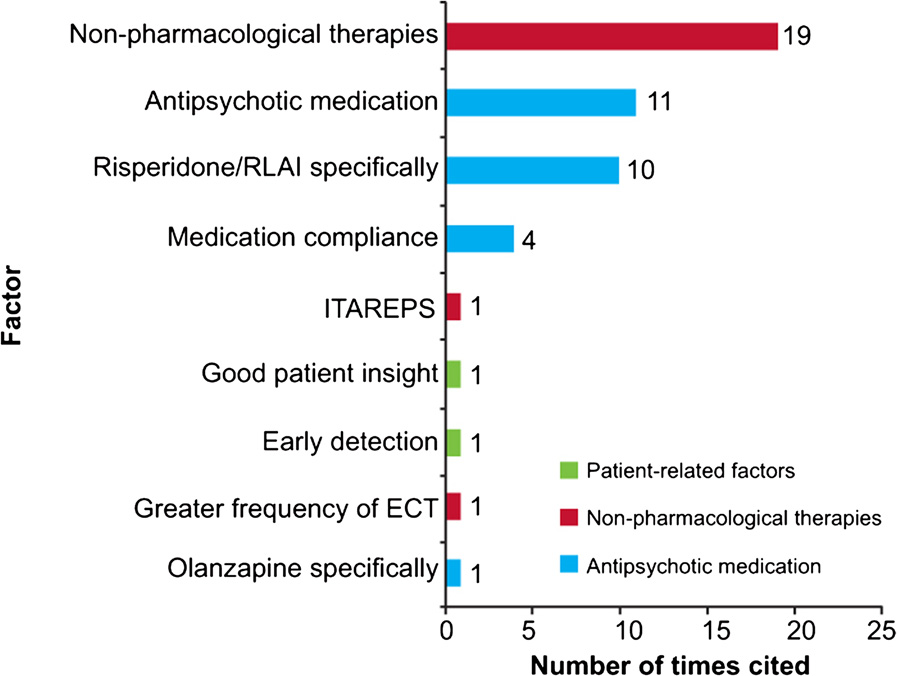
**Factors that may reduce relapse rates.** Individual citations of each factor: a single reference may include citations of more than one factor. The antipsychotic medication category does not include the other pharmacological therapy factors. Non-pharmacological therapies [4-6,11,14-16,27,32,41,42,64],[82,84,92,118-121]; antipsychotic medication [4,6,23,54,81,122-127]; risperidone/risperidone long-acting injectable (RLAI) specifically [22-24,35,65,74,128-131]; medication compliance [25,33,132,133]; Information Technology Aided Relapse Prevention in Schizophrenia (ITAREPS) [52]; good patient insight [134]; early detection [135]; greater frequency of electroconvulsive therapy (ECT) [90]; olanzapine specifically [21].

There were 49 occurrences (in 46 publications) of factors that may reduce the rate of relapse (Figure 4). The potential for antipsychotic therapy to reduce relapse rates was examined in a total of 25 publications. One publication reported the results from a 3-year follow-up of a large cohort of outpatients with schizophrenia taking part in the prospective, observational European Schizophrenia Outpatient Health Outcomes (SOHO) study (*n* = 6516) [21]. In this study, relapse rates were higher in patients who took typical antipsychotics (depot (relative risk (RR) = 1.689; 95% CI = 1.306, 2.184; *p* < 0.0001) or oral (RR = 1.655; 95% CI = 1.316, 2.081; *p* < 0.0001)) than the atypical antipsychotic olanzapine. Differences in the RR for relapse were also observed between olanzapine and other second-generation antipsychotics (SGAs) [21].

Another study showed that patients who were treated with depot antipsychotics had a higher rate of major relapses and hospitalization compared with patients who had not received depot antipsychotics [76]. However, this study primarily examined the outcome of therapy in relation to medication adherence and defined a ‘depot patient’ as one who had received a depot antipsychotic ‘at any time during the study’, which included patients who started therapy with an oral antipsychotic but were later switched to a depot antipsychotic due to poor adherence. Therefore, the extent to which adherence problems caused both the switch to depot antipsychotic therapy and the relapse in these ‘depot patients’ is unclear. Moreover, it was not reported whether relapse occurred prior to, or following, initiation of treatment with the depot antipsychotic [76].

The effect of risperidone long-acting injectable (RLAI) on relapse rates and duration of hospitalization or relapse compared with baseline [22,128] or compared with patients treated with oral antipsychotics was reported in several publications [18,23]. In one *post hoc* analysis of two similarly designed 2-year studies (one RLAI and one oral antipsychotic study), RLAI administration was associated with a relapse rate of 9.3% compared with 42.1% in patients treated with oral antipsychotics (*p* = 0.001) [18]. In a study comparing hospitalization at 1 and 2 years after RLAI initiation, greater decreases from baseline in the number of patients hospitalized and the number and length of hospital stays in patients who continued with RLAI treatment were observed, compared with those who discontinued [24].

Non-pharmacological interventions, such as psychoeducation and cognitive behavioural therapy (CBT), were also commonly reported as factors that may reduce relapse. However, these interventions were evaluated in patients already receiving treatment with antipsychotic medication [4]. Many publications highlighted a ‘trend’ associating therapies such as CBT and psychoeducation with relapse reduction but failed to demonstrate statistically significant effects in observational or naturalistic settings.

## Discussion

The consistent and correct assessment and management of relapse in patients with schizophrenia are vital for clinical practice and important factors for controlled clinical trials. As such, an awareness of the factors that may be associated with increased and decreased rates of relapse should invariably aid clinical practice and benefit patients. However, reporting physicians do not always state how they define relapse; only 62% (87/145) of the final selection of journal articles identified in this literature search stated a definition of relapse. Interestingly, none of the international and national guidelines define relapse, potentially indicating that in clinical practice, a psychiatrist is deemed able to identify a relapsing patient. Alternatively, acutely exacerbated patients may present with a range of signs and symptoms to such a variable degree as to hinder the provision of a unique reliable definition of relapse.

Csernansky and colleagues [17] proposed a set of multifactorial criteria for defining relapse, including hospitalization, and suggested that any single factor could be used as a clinical determinant of relapse. Within the studies identified in this search, many factors were used to define relapse. Hospitalization, usually defined in generic terms of ‘hospitalization’ or ‘psychiatric hospitalization’, was the single factor most commonly used to define relapse and represents a commonly used proxy for examining relapse; however, hospitalization was also one of the search terms used and so may bias the results. In particular, the majority of the retrospective database analyses identified in this literature search specifically investigated hospitalization when conducting their analyses, rather than relapse or other parameters that could potentially be used to define relapse (that may not have been available in the original data source). It is also likely that hospitalization is frequently used to define relapse since it is simple to measure and provides tangible data to analyse. However, schizophrenia is a heterogeneous condition in which a patient might relapse (moderate symptom exacerbation) and not be hospitalized or conversely might be hospitalized for other reasons, such as social or somatic causes, but have relatively stable psychiatric symptoms.

Clinical scales and criteria were also frequently identified in the literature and provide a clinically validated and standardized method of assessment. In clinical studies, where symptoms are measured at baseline and then at set intervals, scales are ideal to characterize patients; however, they can be time-consuming and require additional training to perform since most of them are not intuitive, and are therefore often inconvenient for use in routine clinical practice. Behavioural changes and clinical assessments were least frequently used to define relapse in patients with schizophrenia and were poorly defined in the literature, but are likely to be used in everyday clinical practice. The low frequency of use in clinical studies probably reflects that physician variability may be a significant factor in behavioural and clinical definitions.

Factors that were associated with an increased or decreased risk of relapse included adherence to medication, stress, psychosocial therapies, previous hospitalization/relapse and patient insight. The most prominent factor related to increased risk of relapse was partial/non-adherence to antipsychotic medication [25,76,85]. Indeed, it is well established that treatment with antipsychotic medication can offer an effective option for relapse prevention as well as other beneficial patient outcomes. For instance, in an analysis of a nationwide cohort of 2,588 consecutive patients hospitalized for the first time with a diagnosis of schizophrenia in Finland, 1,496 patients (57.8%) were rehospitalized due to relapse during a mean follow-up period of 2 years; use of any antipsychotic was associated with a lower risk of rehospitalization compared with no use of antipsychotics (Cox model hazard ratio = 0.38, 95% CI = 0.34–0.43; marginal structural model hazard ratio = 0.48, 95% CI = 0.42–0.56) [136].

Increasing gaps in medication intake over 1 year can result in a greater risk of hospitalization (up to a fourfold increase) [26]. The use of continuous medication, through increased adherence to antipsychotic medication or use of medications that give assured delivery, was the most frequently identified factor associated with reduced relapse or hospitalization rates [18,22,23,128]. These findings highlight the current focus on antipsychotic medication in the literature and the importance of monitoring and improving medication adherence in patients with schizophrenia.

While primary research articles concerning RCTs were removed from the initial results for the reasons outlined above (see ‘Methods’), the literature search identified one meta-analysis performed by Leucht et al. [86], covering 17 RCTs, where the heterogeneity of definitions of relapse used mirrored those seen in naturalistic studies. With the exception of RCTs conducted by the same pharmaceutical company, each study used a distinct definition of relapse. Nevertheless, hospitalization, due to an exacerbation of psychotic symptoms, was a key component of most of the methods used to define relapse. While the search was designed to capture definitions of relapse as they relate to routine clinical practice, rather than more selected clinical populations, nevertheless consideration of RCTs of antipsychotic treatments is informative, particularly in terms of their impact on relapse reduction since this is frequently included as one of the study outcomes. Systematic review and meta-analysis of RCTs are often used as a method of comparing the effects of different antipsychotics. One such study suggested that SGAs may have a greater ability to prevent relapse than first-generation antipsychotics (FGAs) [86], but the influence of patient adherence to treatment on this finding is uncertain, and the extent of the difference varied between treatments [137].

A number of meta-analyses have been conducted of relapse rates of depot/long-acting injectable (LAI) antipsychotics compared with oral antipsychotics based on RCTs; however, the study conclusions are not all in agreement. Leucht et al. [138] reported that in studies of 12 months or more comparing depot with oral antipsychotics in schizophrenia, depot formulations reduced relapse significantly, with a relative and absolute risk of 30% and 10%, respectively (RR = 0.70; 95% CI = 0.57, 0.87; number needed to treat (NNT) = 10; 95% CI = 6, 25; *p* = 0.0009). A similar superiority of depot over oral formulations (RR = 0.31; 95% CI = 0.21–0.41 vs RR = 0.46; 95% CI = 0.37–0.57; *p* = 0.03) has been reported in a meta-analysis of RCTs of antipsychotics versus placebo for relapse prevention in schizophrenia [139]. However, a meta-analysis published subsequently by some of the same authors found no superiority of pooled LAIs compared with oral antipsychotics in relapse prevention (studies = 21, *n* = 4950, RR = 0.93; 95% CI = 0.80, 1.08; *p* = 0.35) [140]. The authors speculated that publication bias in older studies (prior to requirements for registration and publication of results of all clinical trials), changing definitions of relapse over time and increasing use of oral SGAs as comparators may all influence the difference in findings between older and more recent RCTs. One interpretation of these differences in findings is that RCTs may over-represent patients with greater adherence to treatment, and with less severe illness compared with the wider population of patients with schizophrenia. Consistent with this assertion, a meta-analysis of mirror-image open studies (pre- and post-introduction of LAI antipsychotics) within subjects showed a strong superiority of LAI antipsychotics in preventing a next hospitalization (16 studies, *n* = 4,066; RR = 0.43; 95% CI = 0.35–0.53; *p* < 0.001, NNT = 3) and in decreasing the number of hospitalizations (15 studies, 6,396 person-years, rate ratio = 0.38; 95% CI = 0.28, 0.51; *p* < 0.001) [141]. This clear superiority was maintained in subgroup analyses of FGA LAI antipsychotics, SGA LAI antipsychotics, studies published before 2000, studies published after 2000, studies reporting intention-to-treat analyses and studies reporting observed cases, with the authors concluding that analyses of naturalistic studies may better represent the clinical population likely to be treated with LAI antipsychotics in routine care.

In non-RCTs identified in the current literature review, non-pharmacological interventions, such as psychoeducation and CBT, were also commonly reported as factors that may reduce relapse and were recommended as important components of schizophrenia management strategies by national guidelines [4].

## Conclusions

In conclusion, this literature search identified numerous factors that have been used to define relapse. Hospitalization was the factor most frequently used to define relapse, and this represents a useful proxy for relapse when reporting in a naturalistic setting. Several factors are reported potentially to increase or decrease the risk of relapse in patients with schizophrenia. Although reliable predictors for relapse have not been identified, observation of these factors may aid the identification of at-risk patients. Continuous antipsychotic medication appears to be one of the most prominently reported factors to reduce the risk of relapse and should be a priority for psychiatrists.

## Competing interests

José M Olivares has participated in regional, national and international advisory boards for Janssen-Cilag, Lilly, Astra-Zeneca and Bristol-Myers Squibb; has been involved in the design and conduct of clinical trials for Janssen-Cilag, Lilly, Astra-Zeneca, Pfizer, Lundbeck, GlaxoSmithKline and Bristol-Myers Squibb; and has received educational grants for research, honoraria and travel support for activities as a consultant/adviser and lecturer/faculty member for Janssen-Cilag, Lilly, Astra-Zeneca, Pfizer, Lundbeck, GlaxoSmithKline, Novartis and Bristol-Myers Squibb. Andreas Schreiner is a shareholder of Johnson & Johnson. Jan Sermon is a full-time employee of Janssen-Cilag NV/SA. Michiel Hemels is a full-time employee of Janssen Health Economics Market Access and Reimbursement, Europe, Middle East and Africa. Andreas Schreiner is a full-time employee of Janssen-Cilag Medical and Scientific Affairs Europe, Middle East and Africa.

## Authors’ contributions

JMO, JS, MH and AS contributed to the development and content of the manuscript and to the interpretation of the search findings and provided guidance on both. All authors read and approved the final manuscript and were responsible for taking the decision to submit the paper for publication.
